# Peri-operative plasma disappearance rate of indocyanine green after coronary artery bypass surgery

**Published:** 2007

**Authors:** Michael Sander, Claudia D Spies, Achim Foer, Doh-Yung Syn, Christian Von Heymann, Herko Grubitzsch

**Affiliations:** Department of Anesthesiology and Intensive Care Medicine, Campus Charité Mitte and Campus Virchow-Klinikum, Charité Universitätsmedizin Berlin, Berlin, Germany; Department of Anesthesiology and Intensive Care Medicine, Campus Charité Mitte and Campus Virchow-Klinikum, Charité Universitätsmedizin Berlin, Berlin, Germany; Department of Anesthesiology and Intensive Care Medicine, Campus Charité Mitte and Campus Virchow-Klinikum, Charité Universitätsmedizin Berlin, Berlin, Germany; Department of Anesthesiology and Intensive Care Medicine, Campus Charité Mitte and Campus Virchow-Klinikum, Charité Universitätsmedizin Berlin, Berlin, Germany; Department of Anesthesiology and Intensive Care Medicine, Campus Charité Mitte and Campus Virchow-Klinikum, Charité Universitätsmedizin Berlin, Berlin, Germany; Department of Cardiovascular Surgery, Campus Charité Mitte, Charité Universitätsmedizin Berlin, Berlin, Germany

## Abstract

Splanchnic ischaemia and hepatic dysfunction are severe complications after coronary artery bypass grafting (CABG) and lead to increased morbidity and mortality. Non-invasive determination of the indocyanine green (ICG) plasma disappearance rate (PDR) offers an opportunity for the early diagnosis of hepato-splanchnic hypoperfusion. The aim of this study was to establish the postoperative time course of the ICG PDR in elective uncomplicated CABG surgery.

After ethical approval and written informed consent, the data of 40 patients were analysed during this prospective study. Measurements of the ICG PDR and cardiac index (CI) in 40 patients undergoing elective CABG surgery were performed immediately after induction of anaesthesia, on admission to the ICU, six hours after admission to the ICU, and on the first postoperative day.

Prior to surgery, baseline ICG PDR was 17.7 %/min (13.6–20.4) and baseline CI was 2.2 l/min/m2 (1.9–2.4). All measurements after surgery showed a significantly higher PDR and cardiac index compared to the baseline measurements. The only patient with prolonged ICU treatment failed to show this increase in ICG PDR, although the CI did increase after surgery.

We established normal values of ICG PDR after uncomplicated CABG surgery. The elevated ICG PDR observed in our patients was assumed to be an effect of an increased hepato-splanchnic blood flow due to an increase in the CI. Patients at risk of hepato-splanchnic hypoperfusion, displaying a missed increase or even a decrease in their ICG PDR after surgery might be at risk of hepatic hypoperfusion and in these selected patients the ICG PDR could serve as a tool to guide therapy or to select patients who might benefit from more invasive devices to monitor hepatosplanchnic perfusion and function.

## Summary

After cardiopulmonary bypass (CPB), up to 20% of patients suffer from transient hepatic dysfunction.^1^ Peri-operative low output, regional perfusion abnormalities as well as underlying pre-operative hepatic diseases are considered to play a crucial role in this. Splanchnic ischaemia is a severe complication after coronary artery bypass grafting (CABG).^2^ In the literature, the reported incidence of splanchnic hypoperfusion leading to surgical intervention was low and ranged between 0.2 and 2%.^3^,^4^ However, mortality in these patients rose as high as 60%.^3^,^4^ Inadequate splanchnic perfusion and oxygenation seems to damage the mucosa of the intestine before any other tissue is compromised.^5^ There is growing evidence that even transient splanchnic hypoperfusion can lead to severe postoperative complications and affect outcome.^6^ Mechanisms leading to this negative impact are ischaemia–reperfusion, bacterial translocation and accompanying immunological cascades resulting in immune paralysis, sepsis and death.^6^-^10^ Therefore, interest is increasing in monitoring splanchnic perfusion in cardiac surgical patients. However, detection of splanchnic hypoperfusion is challenging as there are only a few devices available to gather bedside information in a short period of time.

One of the problems in the past has been the early recognition of patients with impaired hepatic function because standard liver-function tests are neither sensitive nor specific in their identification of patients at risk.^11^,^12^ So, in a considerable number of patients, splanchnic hypoperfusion and hepatic dysfunction remain undetected for too long. Splanchnic hypoperfusion seems to be one of the key factors for developing multi-organ dysfunction syndrome (MODS).^13^,^14^ Inadequate gastrointestinal perfusion and oxygenation seem to damage the mucosa of the intestine. This probably occurs because of a compromised barrier function and diminished perfusion and oxygenation before any systemic sign of hypoperfusion is detected.^15^ Correction of regional oxygenation and perfusion might be of pivotal relevance to reduce endothelial damage and ischaemia–reperfusion episodes and might therefore lower the risk of MODS.

The recent introduction of a new, non-invasive method to measure indocyanine green (ICG) plasma disappearance rate (PDR) using pulse densitometry (LiMON, Pulsion Medical AG, Munich, Germany) offers an opportunity for the early diagnosis of hepatic dysfunction. Clinical data has validated ICG PDR as a marker of hepato-splanchnic function and perfusion.^14^,^16^ A previous study detected a strong association between ICG PDR and outcome in critically ill patients.^17^,^18^

The aim of this study was to establish the postoperative time course of the ICG PDR in elective uncomplicated CABG surgery.

## Methods

Following local ethical committee approval and written, informed consent, 40 patients scheduled for coronary bypass grafting were included in this prospective observational study, according to the principles established in Helsinki.

Diagnosis, surgery and length of ICU stay were documented for each patient. Vital signs, routine laboratory parameters and complications were recorded on a daily basis. Measurement of the plasma disappearance rate of indocyanine green was performed immediately after induction of anaesthesia, on admission to the ICU, six hours after admission to the ICU, and on the first postoperative day. For each measurement, 0.5 mg/kg body weight indocyanine green (Pulsion Medical AG, Munich, Germany) was injected into a central vein. The sensor for measuring the pulse densitrometric decay was placed on the right index finger of the patient and the dye’s decay was analysed with a commercially available monitor (LiMON, Pulsion Medical AG, Munich, Germany). Each measurement was recorded on a laptop for later analysis.

At the same time as the ICG PDR measurements, cardiac index (CI) was determined under stable haemodynamic conditions. All volume substitution was stopped and no changes in vasoactive therapy were allowed during the measurements. The CI was measured by triple injection of 10 ml iced isotone sodium chloride solution into the central venous line of a pulmonary arterial catheter (PAC). The CI was calculated by a commercially available monitor (CI module, Marquette Hellige, Solar 8000, Freiburg, Germany). In case of a deviation of more than 10% in a measurement, five measurements were performed and the highest and lowest were rejected.

After oral pre-medication with 0.1 mg/kg body weight midazolam, general anaesthesia was introduced in all patients. Intravenous induction was performed with etomidate (0.2 mg/kg) and 5 μg/kg fentanil, 0.1 mg/kg pancuronium, followed by a continuous infusion of 5–10 μg/kg/h fentanil, repetition boluses of 0.1 mg/kg midazolam and 0.03 mg/kg pancuronium before the start of cardiopulmonary bypass. Anaesthesia was maintained with 0.6–1 end-tidal volume percent isofluran. All patients were ventilated with an oxygen–air mixture (FiO_2_ 0.5) to maintain an end-tidal pCO_2_ of 35–45 mmHg. Before induction of anaesthesia, haemodynamic monitoring was established with a radial artery catheter for invasive blood pressure monitoring.

Heart rate (HR), arterial blood pressure (systolic and diastolic) and central venous pressure were continuously monitored and recorded (Solar 8000; Marquette Hellige, Freiburg, Germany). Arterial oxygen saturation was continuously monitored by pulse oximetry. Inspired oxygen fraction and end-tidal isoflurane concentration as well as end-tidal CO_2_ were measured (Solar 8000). Additional monitoring in all patients included oesophageal temperature and tidal volume measurements. After orotracheal intubation, a four-lumen central venous catheter (Arrow, Reading, PA) and a thermodilution pulmonary artery catheter (8.5 Fr; Arrow, Reading, PA) were inserted into the right internal jugular vein.

After sternotomy, aprotinin was applied in a dose of 1.5 × 106 IU (total dose of aprotinin was 50 000 KIU/kg body weight including the priming of the CPB). Prior to CPB, 400 IU/kg heparin (Liquemin® Hoffmann-La-Roche, Grenzach-Wyhlen, Switzerland) and additional boluses of 50 IU/kg were given if necessary to maintain an activated clotting time (ACT) of at least 410 s. Routine CPB priming included HES 10%, balanced electrolyte solution and heparin (8 000 IU). CPB was performed under normothermic conditions (blood temperature > 35.5°C) using a membrane oxygenator and centrifugal pump flows adjusted to the calculated cardiac index of 3 l/min/m^2^. During CPB, a venous saturation of over 75% and an MAD above 55 mmHg were aimed at. In case the venous oxygen saturation decreased below 75% and/or the MAD decreased below 55 mmHg, firstly the pump flow was increased and thereafter norepinephrine was administered. Warm intermittent antegrade blood cardioplegia was used.^19^

All data were expressed as median, and 25th and 75th percentiles due to an assumed skewed distribution of data. The intra-group statistical analysis between baseline parameters and during the postoperative course was performed with the Wilcoxon signed rank test. We adjusted for multiple testing with the method of Bonferroni-Holm. The numerical calculations were carried out with SPSS for WINDOWS, version 11.5.1, copyright 1989–2002, SPSS Inc.

## Results

**Fig. 1.**
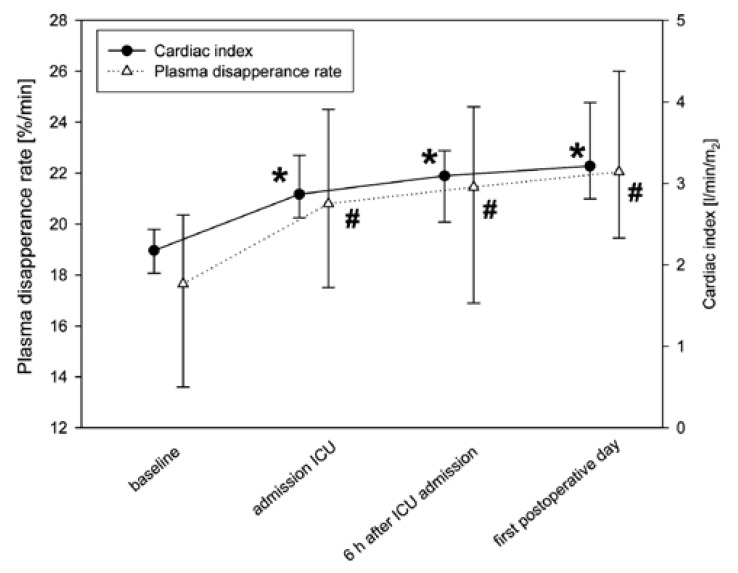
Mean cardiac index and mean PDR ICG prior to and after surgery, *n* = number of analysable ICG PDR measurements; *p < 0.01 compared to baseline cardiac index, ^#^*p* < 0.01 compared to baseline ICG PDR.

During the study period, 160 measurements of ICG PDR were performed in 40 patients undergoing elective CABG surgery. Patients’ basic characteristics are given in Table 1T. Mean number of grafts was three (2–3). Due to motional artefacts, 27 measurements (16.9%) were not analysable, ie, 133 measurements remained for analysis. Baseline ICG PDR prior to surgery was 17.7 %/min (13.6–20.4). All measurements after surgery showed a significantly higher PDR compared to the baseline measurement (Fig. 1).

**Table 1:** Basic Patient Characteristics

	*n*	*Median*	*IQR*
Age (yr)	40	61	56–69
Height (m)	40	1.76	1.72–1.80
Weight (kg)	40	88	79–95
BMI (kg/m^2^)	40	27.8	25.7–31.1
Gender (female/male)	40	12/28	
Pre-operative LVEF (%)	40	50	50−55
ALT baseline (U/l)	40	8	7–32
AST baseline (U/l)	40	25	17−30
Duration of anaesthesia (min)	40	290	255−320
Duration of surgery (min)	40	190	160–215
Duration of CPB (min)	40	70	52–82
Aortic cross-clamp time (min)	40	45	33–58
APACHE II	40	14	9–19

BMI: body mass index; ALT: alanine aminotransferase; AST: aspartate aminotransferase; CPB: cardiopulmonary bypass; LVEF: left ventricular ejection fraction; APACHE II: acute physiology and chronic health evaluation II score.

The baseline cardiac index prior to surgery was 2.2 l/min/m^2^ (1.9–2.4). Likewise, all measurements after surgery confirmed a significantly higher cardiac index compared to the baseline measurement (Fig. 1). Heart rate and mean arterial pressure were also significantly increased after surgery compared to baseline (Table 2). Central venous pressure (CVP) and pulmonary capillary wedge pressure, however, did not increase after surgery (Table 2). Catecholamines were used in 16/40 (40%) patients for weaning from CPB. We found no correlation between intra-operative catecholamine usage and postoperative ICG PDR (*p* = 20.12; *p* = 0.79).

**Table 2:** Haemodynamic Measurements

	*n*	*Median*	*IQR*	p*-value*
Heart rate baseline (1/min)	40	66	57–75	
Heart rate admission ICU (1/min)	40	76	69–87	< 0.01
Heart rate 6h ICU (1/min)	40	74	65–81	< 0.01
Heart rate day 1 ICU (1/min)	40	84	75–91	< 0.01
Mean arterial pressure baseline (mmHg)	40	72	62–80	
Mean arterial pressure admission ICU (mmHg)	40	80	72–92	< 0.01
Mean arterial pressure 6 h ICU (mmHg)	40	80	75–86	0.01
Mean arterial pressure day 1 ICU (mmHg)	40	79	72–89	0.03
Central venous pressure baseline (mmHg)	40	12	9–15	
Central venous pressure admission ICU (mmHg)	40	10	7–13	0.15
Central venous pressure 6 h ICU (mmHg)	40	10	7–13	0.02
Central venous pressure day 1 ICU (mmHg)	40	10	7–14	0.09
PCWP baseline (mmHg)	40	12	9–14	
PCWP admission ICU (mmHg)	40	13	10–15	0.67
PCWP 6h ICU (mmHg)	40	12	10–14	0.56
PCWP day 1 ICU (mmHg)	40	12	9−14	0.31

ICU: intensive care unit; PCWP: pulmocapillary wedge pressure.

Surgery and CPB management was uncomplicated in all but one patient (Table 3) who demonstrated an acute biventricular failure after weaning from CPB, requiring implantation of an intra-aortic balloon pump (IABP). ICU treatment was uncomplicated in most cases. Thirty-six patients were transferred to the intermediate-care unit the day after surgery. Two patients were transferred after two days of ICU treatment and one patient after four days due to pulmonary dysfunction, requiring CPAP treatment. The patient with acute cardiac failure after CPB developed postoperative pneumonia and acute renal failure, requiring continuous veno-venous renal replacement (CVVH) therapy and needed 52 days of ICU treatment before being transferred to the intermediate-care unit. Median ICU treatment time was one day (1–1). Patients were mechanically ventilated for a mean treatment time of 10 hours (8–13).

**Table 3:** Intra-And Postoperative Measurements

	*n*	*Median*	*IQR*
CI during CPB (l/min/m^2^)	40	3.2	3.0–3.6
Temperature during CPB (°C)	40	35.7	35.0–36.0
Cumulative norepinephrine dosage during CPB (mg)	40	0.08	0.06–0.09
Dopamine dosage for weaning from CPB (μg/kg/min)	40	1.0	0.0–3.0
Number of patients with catecholamines for weaning from CPB (*n*)	40	16	
Patients with IABP for weaning from CPB (*n*)	40	1	
Patients with acute cardiac failure during weaning from CPB (*n*)	40	1	
Urine volume during CPB (ml)	40	156	97–280
Urine volume during first 24 h ICU (ml)	40	2810	2310−3478
Lactate during CPB (mmol/l)	40	0.8	0.6−1.1
Lactate admission ICU (mmol/l)	40	1.4	1.2–1.9
Lactate after 6 h ICU treatment (mmol/l)	40	1.9	1.3–2.5
Lactate after 1 day ICU treatment (mmol/l)	40	1.4	1.0–2.1

CI: cardiac index; CPB: cardiopulmonary bypass; IABP: intra-aortic balloon pump; ICU: intensive care unit.

Arterial lactate levels were low throughout the whole study period (Table 3). Maximum levels of postoperative ALT and AST were 22 U/l (15–38) and 27 U/l (19–39), respectively, and therefore within normal limits.

**Fig. 2.**
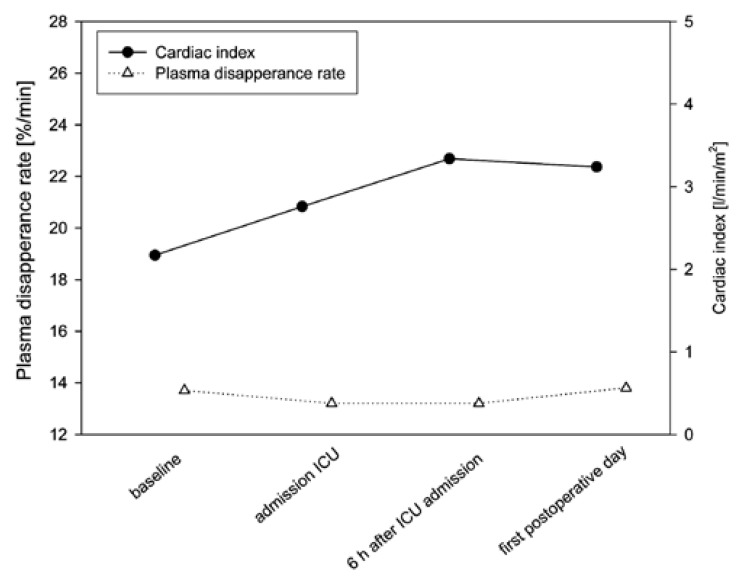
Cardiac index and ICG PDR of the patient with prolonged need for intensive care treatment.

The patient with the prolonged ICU treatment did not show an increase in his ICG PDR, although the CI did increase after surgery (Fig. 2). This patient had prolonged time to first bowel movement and increased levels of transaminases (maximum ALT and AST during ICU treatment was 121 IU/l and 145 IU/l, respectively). Also, postoperative lactate levels were markedly increased (maximum six hours after surgery, 8.3 mmol/l).

## Discussion

This study evaluated the time course of ICG PDR peri-operatively in uncomplicated CABG surgery patients. The most important finding was that patients with uncomplicated surgery showed a significant increase in their postoperative ICG PDR compared to pre-operative baseline measurements. This finding might indicate that splanchnic perfusion was increased and hepatosplanchnic function was not severely compromised after surgery in these patients with an uncomplicated postoperative course. In line with this finding was that we did not observe a significant increase in transaminases after surgery. This study therefore established normal values of ICG PDR after uncomplicated CABG surgery. This provides the opportunity to identify future patients with abnormally low values of ICG PDR, possibly due to severely decreased cardiac function, who might be in danger of complications due to splanchnic hypoperfusion.

ICG PDR values have been validated predominantly in critically ill patients and in patients after liver transplantation.^17^,^18^,^20^ Kimura *et al.* showed that extremely low ICG PDR or failure to improve PDR were signs of poor outcome.^17^ In patients after liver transplantation, the ICG PDR is used to detect early transplant failure and to monitor transplant function.^20^ A recent study also found an increased PDR after CABG surgery.^21^ In this study, however, global perfusion indices and the corresponding perioperative PDR after CABG surgery have not been evaluated.^21^

Surgery and, even more specifically, cardiac surgery leads to a postoperative inflammatory response.^22^ This response is characterised by elevated pro-inflammatory cytokines, leukocytosis, fever and increased cardiac output.^15^,^22^ This increased cardiac output was also observed in our patients. In part, this increase was also achieved by optimised volume management and catecholamine therapy. However, splanchnic perfusion does not always parallel global perfusion^23^ and is reported to be compromised in some patients after CPB surgery.^12^,^24^ In a study by Sakka *et al.*, volume loading and optimisation of global haemodynamic parameters did not always lead to an increase in splanchnic perfusion.^23^ Splanchnic hypoperfusion is associated with increased morbidity and mortality after CABG surgery.^3^,^4^,^25^ As shown by our group, even patients with an uncomplicated and clinically uneventful postoperative course are subject to increased hepato-splanchnic oxygen consumption and therefore might experience clinically silent splanchnic hypoperfusion.^15^ Therefore, an easily obtainable and clinically reliable marker of hepato-splanchnic function and perfusion would be a desirable device to measure peri-operatively at repeated time intervals.

We observed in our study an increase in ICG PDR and cardiac output after surgery. Therefore we suggest that the higher PDR might be the effect of an increased splanchnic blood flow due to an increase in CI in uncomplicated patients. However, as we did not measure hepatic blood flow directly in our patients, we cannot prove that hepatic perfusion improved after surgery. Nevertheless, the increased ICG PDR must have been an effect of either increased liver function or perfusion.

ICG, a water-soluble tricarboncyanine, is irreversibly removed by hepatocytes in a flow-dependent manner, into the bile.^26^ Relevant extra-hepatic elimination pathways do not exist. As demonstrated by our group and others, liver-function tests such as the MEGX test are neither changed nor impaired when compared to preoperative values.^15^,^27^ Therefore it seems reasonable to impute that liver function is not enhanced after CPB for elective CABG. The postoperative increased ICG PDR seen in our patients was therefore assumed to be the effect of an increased hepatic blood flow, which was supported by an increase in CI.

Arterial lactate levels were low in our patients after surgery. This finding is in line with the assumption of insignificantly compromised hepatic function in the patients studied. Lactate is voided into the systemic circulation after anaerobic glycolysis due to hypoperfusion and inadequate oxygen supply.^28^,^29^ An important source of lactate after cardiac surgery with CBP is reported to be the gastrointestinal tract.^29^-^31^ Lactate is cleared predominantly by the liver. The slightly elevated levels of lactate on admission to the ICU and during the subsequent measurements may indicate that adequate hepatic metabolism has to be assumed, which depends on normal hepatic blood flow perioperatively.^31^

Routine clinical laboratory parameters did not show any signs of hepatic dysfunction in our patients. Even maximum levels of ALT and AST were within the normal range in all patients. However, standard liver function tests are neither sensitive nor specific in their identification of patients with impaired hepatic function.^12^,^32^ Therefore, in quite a number of patients, splanchnic hypoperfusion and hepatic dysfunction remain undetected for too long. Significant elevations of ALT and AST point to structural damage to the liver, but this is noticed very late after the onset of hepato-splanchnic hypoperfusion. Therefore detection of elevated transaminases cannot be used to prevent damage to the hepato-splanchnic system – it can only be used to limit damage. Conversely, the determination of ICG PDR levels can help identify patients at risk of hepatic hypoperfusion and dysfunction at an earlier stage.

A limitation of this study was that we could not comment on the predictive capacity of ICG PDR for complications after CABG surgery as only one patient in this study developed severe complications in the postoperative period. However, this was not the aim of the study. The aim was to establish a normal range of the time course of ICG PDR after uncomplicated CABG surgery. In these patients, splanchnic hypoperfusion is a rare event.

Even though ICG PDR did not increase after surgery in one patient, we cannot generalise this finding and draw conclusions about an association between ICG PDR and the development of postoperative complications. Nevertheless, in patients with a missed increase or even a decrease in their postoperative ICG PDR, intervention might be considered, such as anti-oxidative treatment or vasoactive therapy which is thought to generate a better splanchnic perfusion.^33^,^34^

Another limitation was the relatively high number of motional artefacts of the non-invasive sensor used throughout this study. These were, however, mostly not due to motion of the patient, but to artefacts caused by the surgeons or by movement of the operating table.

In conclusion, we established the normal time course of ICG PDR after uncomplicated CABG surgery. This provides the opportunity to identify future patients with abnormally low ICG PDR, possibly due to severely decreased cardiac function, who might be in danger of complications due to splanchnic hypoperfusion. We assumed that in uncomplicated surgery, the increase in ICG PDR was an effect of increased splanchnic blood flow. In these patients, the increase in global cardiac output was paralleled by an increase in splanchnic blood flow.

However, in some patients there seemed to be an increase in global cardiac output, which was not coupled to an increase in PDR. These patients may have been at risk of hepato-splanchnic hypoperfusion or ischaemia or there might have been a decrease of splanchnic perfusion in these patients. Sequential assessment of ICG PDR may identify patients at risk. However, the validity of ICG PDR to detect hepatic hypoperfusion should be assessed in future trials. In particular, patients with a high risk of splanchnic hypoperfusion (eg, in patients with compromised cardiac function) may benefit from advanced monitoring of the hepato-splanchnic system during and after cardiac surgery.
